# Mitochondrial-related hub genes in dermatomyositis: muscle and skin datasets-based identification and *in vivo* validation

**DOI:** 10.3389/fgene.2024.1325035

**Published:** 2024-02-08

**Authors:** Shuo Wang, Yiping Tang, Xixi Chen, Siyuan Song, Xi Chen, Qiao Zhou, Li Zeng

**Affiliations:** ^1^ School of Medicine, University of Electronic Science and Technology of China, Chengdu, China; ^2^ Department of Internal Medicine, Sichuan Academy of Medical Science and Sichuan Provincial People’s Hospital, University of Electronic Science and Technology of China, Chengdu, China; ^3^ Department of Rheumatology and Immunology, Sichuan Academy of Medical Science and Sichuan Provincial People’s Hospital, University of Electronic Science and Technology of China, Chengdu, China; ^4^ Baylor College of Medicine, Houston, TX, United States; ^5^ Children’s Nutrition Research Center, Department of Pediatrics, Baylor College of Medicine, Houston, TX, United States; ^6^ Clinical Immunology Translational Medicine Key Laboratory of Sichuan Province, Sichuan Provincial People’s Hospital, University of Electronic Science and Technology of China, Chengdu, China; ^7^ Department of Neurology, Sichuan Academy of Medical Science and Sichuan Provincial People’s Hospital, University of Electronic Science and Technology of China, Chengdu, China

**Keywords:** dermatomyositis, mitochondria, bioinformatic analysis, IFI27, CMPK2, LAP3, immune landscape

## Abstract

**Background:** Mitochondrial dysfunction has been implicated in the pathogenesis of dermatomyositis (DM), a rare autoimmune disease affecting the skin and muscles. However, the genetic basis underlying dysfunctional mitochondria and the development of DM remains incomplete.

**Methods:** The datasets of DM muscle and skin tissues were retrieved from the Gene Expression Omnibus database. The mitochondrial related genes (MRGs) were retrieved from MitoCarta. DM-related modules in muscle and skin tissues were identified with the analysis of weighted gene co-expression network (WGCNA), and then compared with the MRGs to obtain the overlapping mitochondrial related module genes (mito-MGs). Subsequently, differential expression genes (DEGs) obtained from muscle and skin datasets were overlapped with MRGs to identify mitochondrial related DEGs (mito-DEGs). Next, functional enrichment analysis was applied to analyze possible relevant biological pathways. We used the Jvenn online tool to intersect mito-MGs with mito-DEGs to identify hub genes and validate them using reverse transcription quantitative polymerase chain reaction (RT-qPCR) and immunohistochemistry staining. In addition, we evaluated immune infiltration in muscle and skin tissues of DM patients using the one-sample gene set enrichment analysis (ssGSEA) algorithm and predicted potential transcription factor (TF) -gene network by NetworkAnalyst.

**Results:** The WGCNA analysis revealed 105 mito-MGs, while the DEG analysis identified 3 mito-DEGs. These genes showed functional enrichment for amino acid metabolism, energy metabolism and oxidative phosphorylation. Through the intersection analysis of the mito-MGs from the WGCNA analysis and the mito-DEGs from the DEG set, three DM mito-hub genes (*IFI27*, *CMPK2*, and *LAP3*) were identified and validated by RT-qPCR and immunohistochemistry analysis. Additionally, positive correlations were observed between hub genes and immune cell abundance. The TF-hub gene regulatory network revealed significant interactions involving ERG, VDR, and ZFX with *CMPK2* and *LAP3*, as well as SOX2 with *LAP3* and *IFI27*, and AR with *IFI27* and *CMPK2*.

**Conclusion:** The mito-hub genes (*IFI27*, *CMPK2*, and *LAP3*) are identified in both muscles and skin tissues from DM patients. These genes may be associated with immune infiltration in DM, providing a new entry point for the pathogenesis of DM.

## 1 Introduction

Dermatomyositis (DM) is a rare autoimmune disorder that affects both the skin and muscles. It is characterized by the presence of auto-antibodies targeting various cellular components, including nuclear and cytoplasmic proteins, leading to inflammation, tissue damage, and muscle weakness (Lundberg et al., 2021). Although the molecular and pathophysiological mechanisms of DM remain unclear, it is believed that gene, environmental and immunological factors play important roles (O'Connell and LaChance, 2021). In DM, T cells, dendritic cells and B cells, and overproduction of type-I interferons are implicated in the pathogenesis of the disease (Kao et al., 2011).

Mitochondria are vital organelles responsible for energy production via oxidative phosphorylation (OXPHOS) and are involved in various cellular processes, including calcium homeostasis, reactive oxygen species (ROS) generation, apoptosis and the innate immune response (Balaban et al., 2005). Several studies have shown that mitochondrial dysfunction is crucial to DM pathogenesis (Alhatou et al., 2004; Varadhachary et al., 2010; Gonzalez-Chapa et al., 2023). DM patients exhibit impaired OXPHOS function, which leads to a reduction in ATP production and an increase in ROS accumulation (Meyer et al., 2017). They also have an increased recovery half-time of phosphocreatine and adenosine diphosphate after exercise (Yang et al., 2015). These abnormalities may contribute to muscle fiber damage, inflammation, and skin manifestations in DM (Lightfoot et al., 2015). Additonally, one of the prominent features of DM is perifascicular atrophy, which is caused by microangiopathy and ischemia within affected muscle tissues (Woo et al., 1988b), and mitochondrial dysfunction has been implicated in it through various mechanisms. For instance, impaired mitochondrial calcium homeostasis can lead to endothelial dysfunction and abnormal angiogenesis. ROS produced by dysfunctional mitochondria can promote oxidative stress and endothelial damage, further exacerbating microvascular alterations (Moos et al., 2021). Moreover, mitochondrial dysfunction may influence the innate immune system by releasing damage-associated molecular patterns (DAMPs) and mitochondrial DNA (mtDNA) into the cytoplasm (Lyu et al., 2023). As a result, innate immune signaling pathways, such as the stimulator of interferon genes (STING) pathway, are activated. This activation ultimately results in the production of pro-inflammatory cytokines and type I interferons, which further perpetuates the autoimmune response in DM (Fang et al., 2016).

Recent research has focused on specific mitochondrial genetic mutations associated with DM pathogenesis. MtDNA variants, especially in the mitochondrial displacement loop, such as 16304T/C and 16519T/C, have been found in DM muscle biopsies (Lax et al., 2011). The latter is also associated with positive antinuclear antibody status as well as interleukin-2 production, indicating a potential link between somatic mutations and DM development (Zhao Y. et al., 2022). Furthermore, mutations in nuclear gene *POLG1*, which is responsible for the synthesis and repair of the mitochondrial genome, has also been identified in DM patients (Lax et al., 2011; Singh et al., 2015). These genetic alterations may disrupt mitochondrial function and trigger immune responses that contribute to the inflammatory process observed in DM. However, previous studies on mtDNA in DM patients have primarily focused on single or a few genes, and there has been limited exploration of the overall genetic background.

**FIGURE 1:**
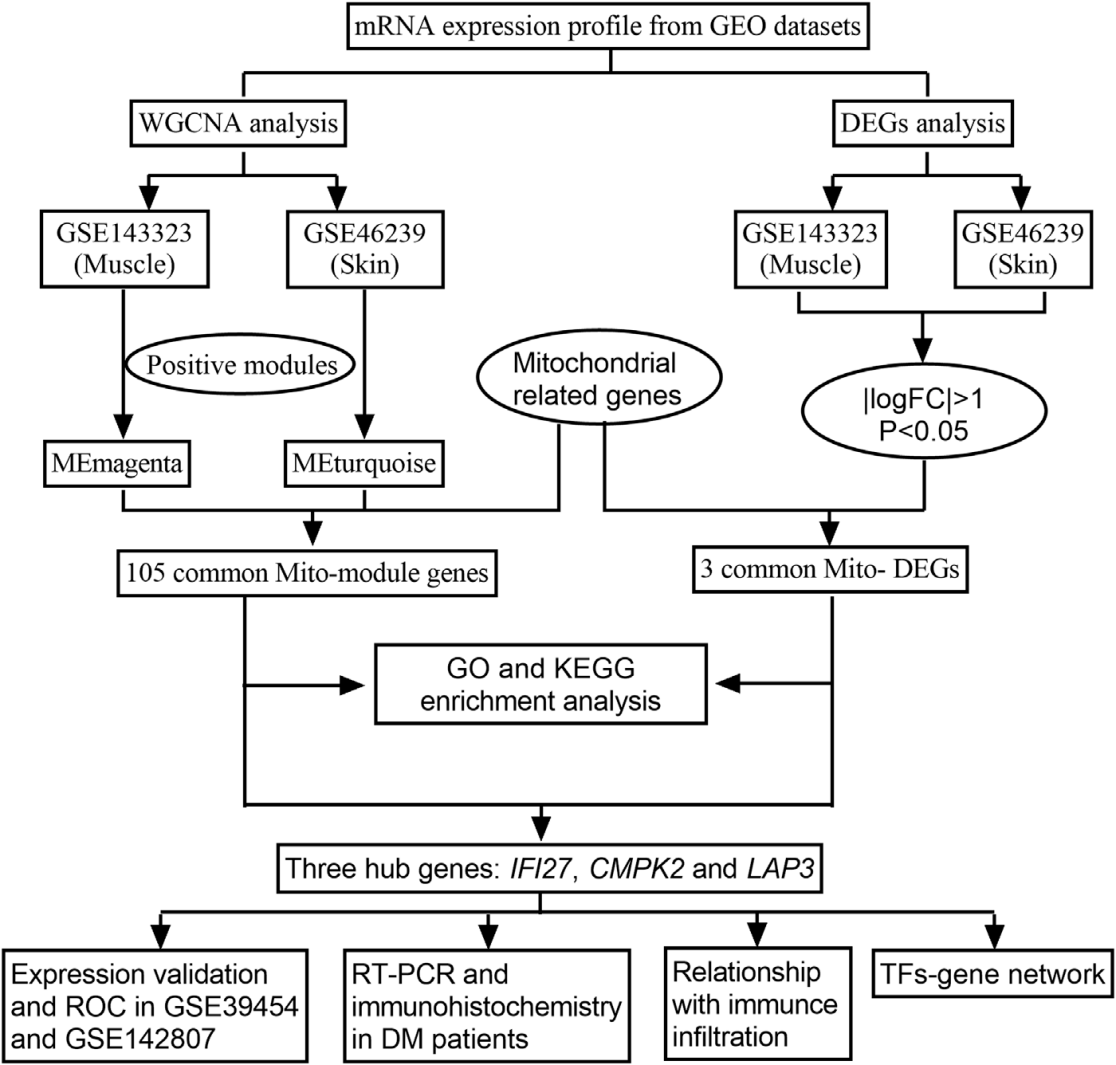
The research flowchart.

The emergence of bioinformatics and high-throughput sequencing has enabled researchers to efficiently scrutinize data from a multitude of genes, thereby providing profound insights into disease pathogenesis at the transcriptional level. Utilizing these approaches, our study aimed to comprehensively analyze the transcriptional profiles of mitochondrial-related genes in DM. We harnessed the potential of weighted gene coexpression network analysis (WGCNA) to identify mitochondrial-related module genes (mito-MGs) and analyzed differentially expressed genes to screen out mitochondrial-related DEGs (mito-DEGs) in DM muscle and skin tissues. Our study integrated an in-depth bioinformatic approach with *in vivo* validation, representing the first investigation into MRG abnormalities in DM from a transcriptional perspective, as illustrated in Figure 1. Through this comprehensive approach, we aim to provide profound insights into the pathogenesis of DM and shed light on potential therapeutic targets.

## 2 Materials and methods

### 2.1 Data source

We searched through the GEO databank for DM gene expression datasets that included human skeletal muscle and skin samples, using the keyword “dermatomyositis.” Four datasets were downloaded: GSE143323, GSE39454, GSE46239 and GSE142807. The former two datasets were derived from muscle tissues, while the latter was generated from skin tissues. GSE143323 and GSE46239 served as the test cohorts, while GSE39454 and GSE142807 served as the validation cohorts. A summary of the four datasets can be found in Table 1. In addition, 1,136 MRGs were obtained from the public database MitoCarta 3.0 with the duplicates removed (http://www.broadinstitute.org/mitocarta) (Rath et al., 2021).

**TABLE 1:** Dermatomyositis related GEO datasets.

	GSE number	Platform	Samples	Organism	Source types	Group
1	GSE143323	GPL21290	39 patients and 20 controls	*Homo sapiens*	Muscle	Discovery
2	GSE46239	GPL570	48 patients and 4 controls	*Homo sapiens*	Skin	Discovery
3	GSE39454	GPL570	8 patients and 5 controls	*Homo sapiens*	Muscle	Validation
4	GSE142807	GPL17692	43 patients and 5 controls	*Homo sapiens*	Skin	Validation

GEO, gene expression omnibus.

### 2.2 WGCNA analysis and identification of mitochondrial-related module genes (mito-MGs)

To obtain DM-related module genes, the WGCNA package in R (Langfelder and Horvath, 2008) was utilized to the datasets GSE143323 and GSE46239 to construct the gene co-expression networks. Detailed procedures can be found in our previous study (Xie et al., 2022). Firstly, clustering analysis was performed on all samples to eliminate outliers. Three samples were excluded from GSE143323 due to poor heterogeneity, while there were no exclusions from GSE46239 due to good heterogeneity (Supplementary File S1). A scale-free topology was then constructed based on an appropriate soft threshold power. After that, modules were identified based on hierarchical clustering, and the eigengene was calculated. In total, 10 and 19 co-expression modules were identified from muscle and skin tissues, respectively. Pearson correlation analysis was used to determine the relationship between the disease phenotype and the modules. Modules with the highest positive correlations were categorized as DM-related modules, and the genes in these modules were defined as the DM-related module genes. By intersecting DM-related module genes with MRGs,a total of 105 mito-MGs were obtained.

### 2.3 DEG analysis and identification of mito-DEGs

DEG analysis was performed on muscle and skin samples from the datasets GSE143323 and GSE46239 with the R package “limma.” The DEGs were identified based on the screening criteria of Log2|fold change (FC)|>1 and a *p*-value < 0.05. The resulting DEGs were displayed with heatmap and Volcano Plot. Furthermore, the mito-DEGs were obtained by intersecting the DEGs from the muscle and skin datasets with the MRGs using a Venn Diagram.

### 2.4 Functional enrichment analysis

Enrichplot and clusterProfiler packages in R were used to annotate Kyoto Encyclopedia of Genes and Genomes (KEGG) and Gene Ontology (GO). “ggplot2” was used to visualize the top ten terms/pathways.

### 2.5 Identification and validation of DM mito-hub genes

Three hub genes were identified by intersecting 105 mito-MGs obtained from WGCNA with three mito-DEGs using the Jvenn online tool. Subsequently, the expression of these mito-hub genes was validated in four datasets using GraphPad Prism software (version 9.3). A Student’s nonparametric t-test was conducted to compare the two groups, with a significance level set at *p* < 0.05. Additionally, the diagnostic capability assessment was performed on the three hub genes by calculating receiver operating characteristic (ROC) curves with “pROC” in R.

### 2.6 RT-qPCR analysis

Between 2021 and 2022, a total of 9 DM patients from the department of rheumatology and immunology in our hospital were included as the experimental group. Each patient fulfilled Bohan and Peter criteria and had DM confirmed by skin/muscle biopsy (Singh et al., 2015). Meanwhile, 11 healthy subjects were selected as the control group. One milliliter of peripheral blood was obtained from each subject as a study sample. We conducted the study in accordance with the Declaration of Helsinki and the Ethics Committee of our hospital approved it. In addition, all subjects provided informed consent by signing the necessary forms. Total RNA from peripheral blood was isolated using TransZol reagent (TransZol Up Plus RNA Kit, TransGen Biotech, Beijing) and reverse transcribed (TransScript All-in-One kit, TransGen Biotech, Beijing). The RT-qPCR analysis was conducted on an ABI Step One Plus instrument ultilizing TransStart Top Green qPCR SuperMix (TransGen Biotech, Beijing) as previously reported (Zeng et al., 2023). In brief, individual tubes were loaded with 0.2 µM of both forward and reverse primers, 0.4 µL Passive Reference Dye, 10 µL SuperMix buffer, and 100 ng cDNA templates. The total volume in each tube was adjusted to 20 µL using RNase-free water (TransGen Biotech, Beijing). PCR activation initiated at 94°C for 30 s, succeeded by 40 cycles of denaturation at 94°C for 5 s and extension at 60°C for 30 s. Triplicate reactions were performed, and changes in the target gene’s expression were normalized to GAPDH levels using the 2^–△△Ct^ method. All primer sequences are presented in Supplementary File S2.

### 2.7 Histological and immunohistochemistry analysis

Histological and immunohistochemical analyses were conducted on muscle tissues obtained from 10 DM patients and 5 control subjects. The specimens were promptly frozen by immersion in liquid nitrogen-cooled isopentane. The frozen sections underwent staining procedures, including Haematoxylin and Eosin (H&E) and NADH tetrazolium reductase (NADH-TR) staining. For immunohistochemistry, the following primary antibodies were applied using standard protocols: anti-human IFI27 (1:50; Affinity, China), anti-human CMPK2 (1:50; Proteintech, United States), and anti-human LAP3 (dilution 1:50; Proteintech, United States). Immunohistochemistry staining was performed utilizing the Novolink Polymer DS kit (Leicabiosystems) following the manufacturer’s guidelines.

### 2.8 Assessment of immune cell abundance in muscle and skin tissues of DM

We conducted an assessment of the level of immune infiltration in muscle as well as skin tissue of DM patients using the single-sample gene set enrichment analysis (ssGSEA) algorithm implemented in the R package. Boxplots were utilized to display the differential expressions of the immune infiltrating cells. Furthermore, a Spearman correlation analysis was performed using the “ggplot2” package to investigate the correlation between immune infiltration and the hub genes.

### 2.9 Prediction of transcription factors (TFs) gene regulatory network

The interactions between the hub genes and transcription factors (TFs) were established utilizing the NetworkAnalyst tool (v2019; https://www.networkanalyst.ca/). Subsequently, Cytoscape was applied to visualize the hub genes and TFs.

## 3 Results

### 3.1 Identification of mito-MGs in muscle and skin through WGCNA analysis

**FIGURE 2:**
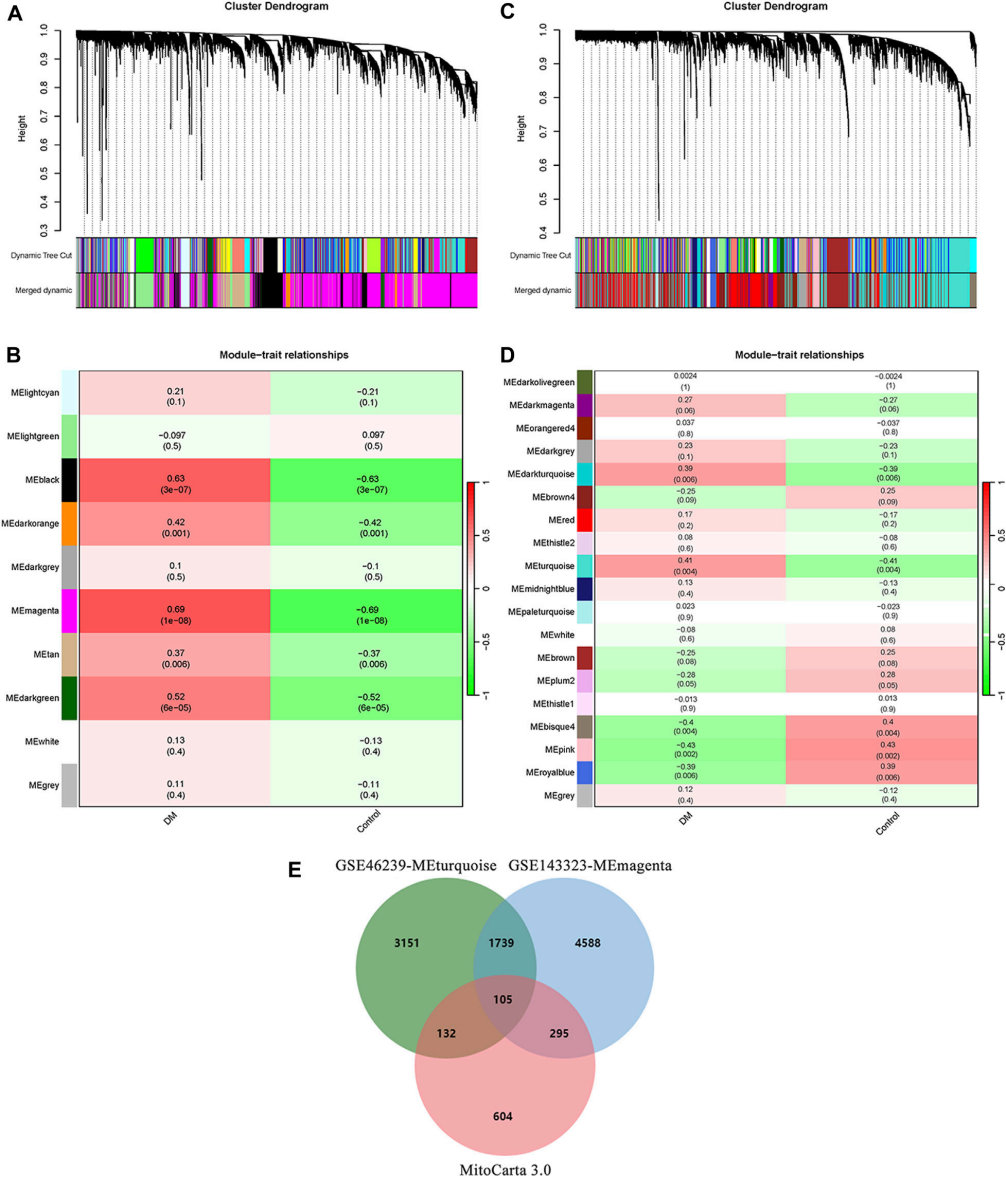
Identification of DM-related module and screening of mito-MGs in muscle and skin tissues from the dataset GSE143323 and GSE46239 via WGCNA analysis. **(A)** Gene cluster dendrogram identifying 10 co-expression modules in GSE143323 dataset (muscle). Co-expression modules are represented by the colors at the bottom of each branch. Gene similarity coefficients are displayed along the y-axis (height). **(B)** Module-trait relationship construction in GSE143323 dataset. The matrix shows correlation coefficients and their corresponding *p* values (in brackets) between modules on the y-axis and disease phenotype on the x-axis. The MEmagenta module was strongly correlated with DM in muscle tissue. **(C)** Gene cluster dendrogram identifying 19 co-expression modules in GSE46239 dataset (skin). **(D)** Module-trait relationship construction in GSE46239 dataset. The MEturquoise module was strongly correlated with DM in skin tissue. **(E)** Venn diagram showing the intersections of genes among MEmagenta, MEturquoise and MRGs. DM, dermatomyositis; mito-MGs, mitochondrial-related module genes. MRGs, mitochondrial-related genes.

To establish scale-free co-expression networks and identify module genes related to DM in muscle and skin tissues, we employed the R package WGCNA to analyze the GSE143323 and GSE46239 datasets. In the GSE143323 dataset, consisting of muscle tissues from 39 DM patients and 20 controls, the DM-related module was identified (Figure 2). Initially, we selected a value of β = 7 to construct a scale-free network for GSE143323 (Supplementary File S3A). Subsequently, module merging yielded 10 gene modules (Figure 2A). A Pearson correlation heatmap was generated to visualize the module-trait relationships based on the correlation coefficient calculated between each module and the phenotype of interest. Among these modules, MEmagenta (*cor* = 0.69; *p* = 1e-8) exhibited the strongest positive correlation with DM and was thus designated as the DM-related module in muscle tissue (Figure 2B). Similarly, in the GSE46239 dataset, which included skin tissue samples from 48 DM patients and 4 healthy controls, we selected a soft power of β = 3 to construct the scale-free network (Supplementary File S3B). A total of 19 gene modules were identified, with MEturquoise displaying the highest correlation with DM (*cor* = 0.41; *p* = 0.004) (Figures 2C, D). The genes within MEturquoise and MEmagenta were subsequently intersected with a set of 1,136 MRGs, resulting in 105 mito-MGs (Figure 2E).

**FIGURE 3:**
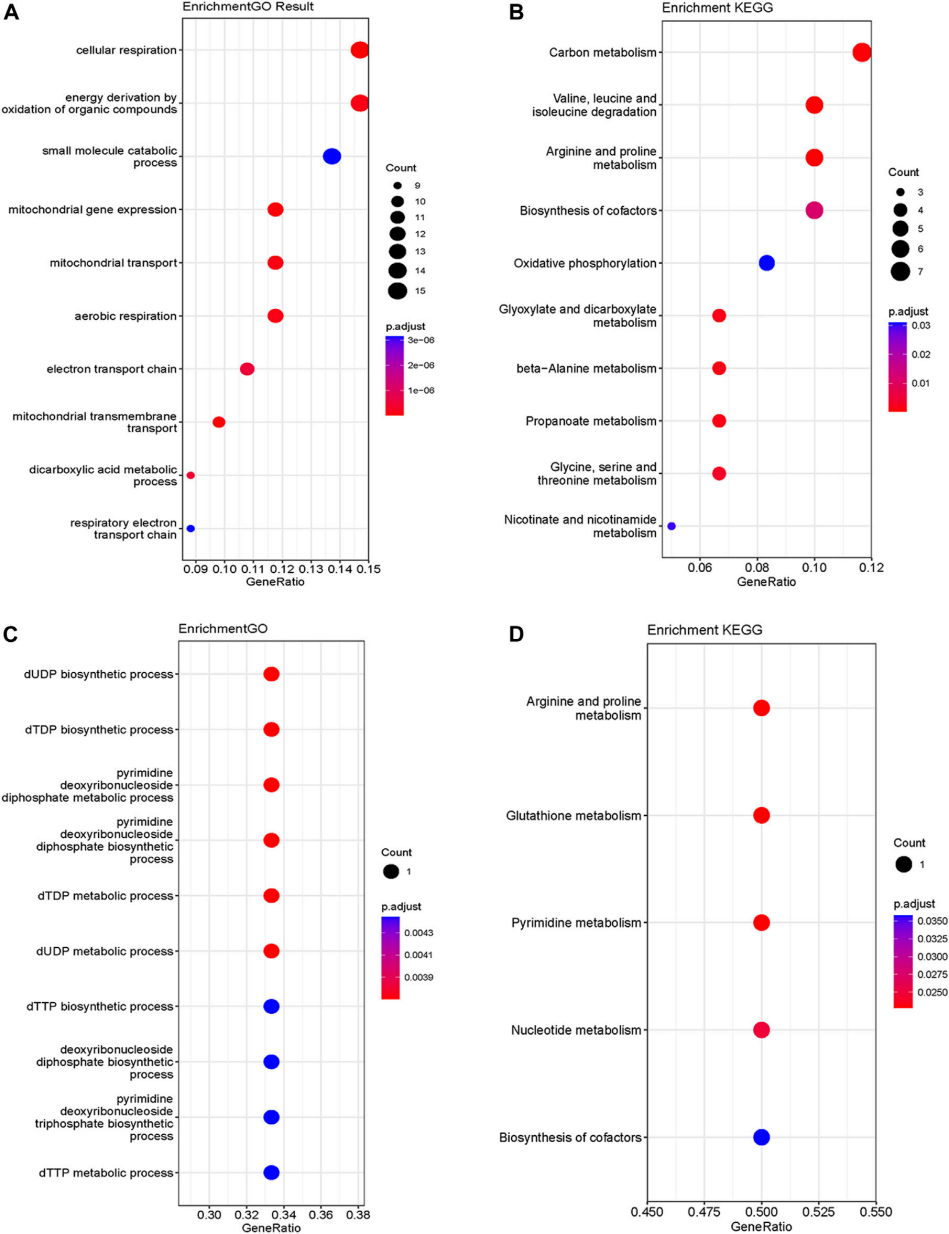
GO **(A)** and KEGG **(B)** functional enrichment analysis of mito-MGs. GO **(C)** and KEGG **(D)** functional enrichment analysis of mito-DEGs. GO, Gene Ontology; KEGG, Kyoto Encyclopedia of Genes and Genomes. Mito-MGs, mitochondrial-related module genes. Mito-DEGs, mitochondrial-related differentially expressed genes.

The analysis of functional enrichment provided insight into the possible biological functions of these genes. In a Gene Ontology (GO) analysis, these genes were primarily associated with cellular respiration, energy derivation by oxidation of organic compounds and mitochondria (Figure 3A). In a KEGG analysis, the gene set exhibited prominent enrichment in amino acid metabolism pathways (e.g., Valine, leucine and isoleucine degradation, Arginine and proline metabolism, Glycine, serine and threonine metabolism). Additionally, pathways related to OXPHOSand energy metabolism were also significantly enriched (Figure 3B).

### 3.2 Identification of mito-DEGs in muscle and skin via DEG set

**FIGURE 4:**
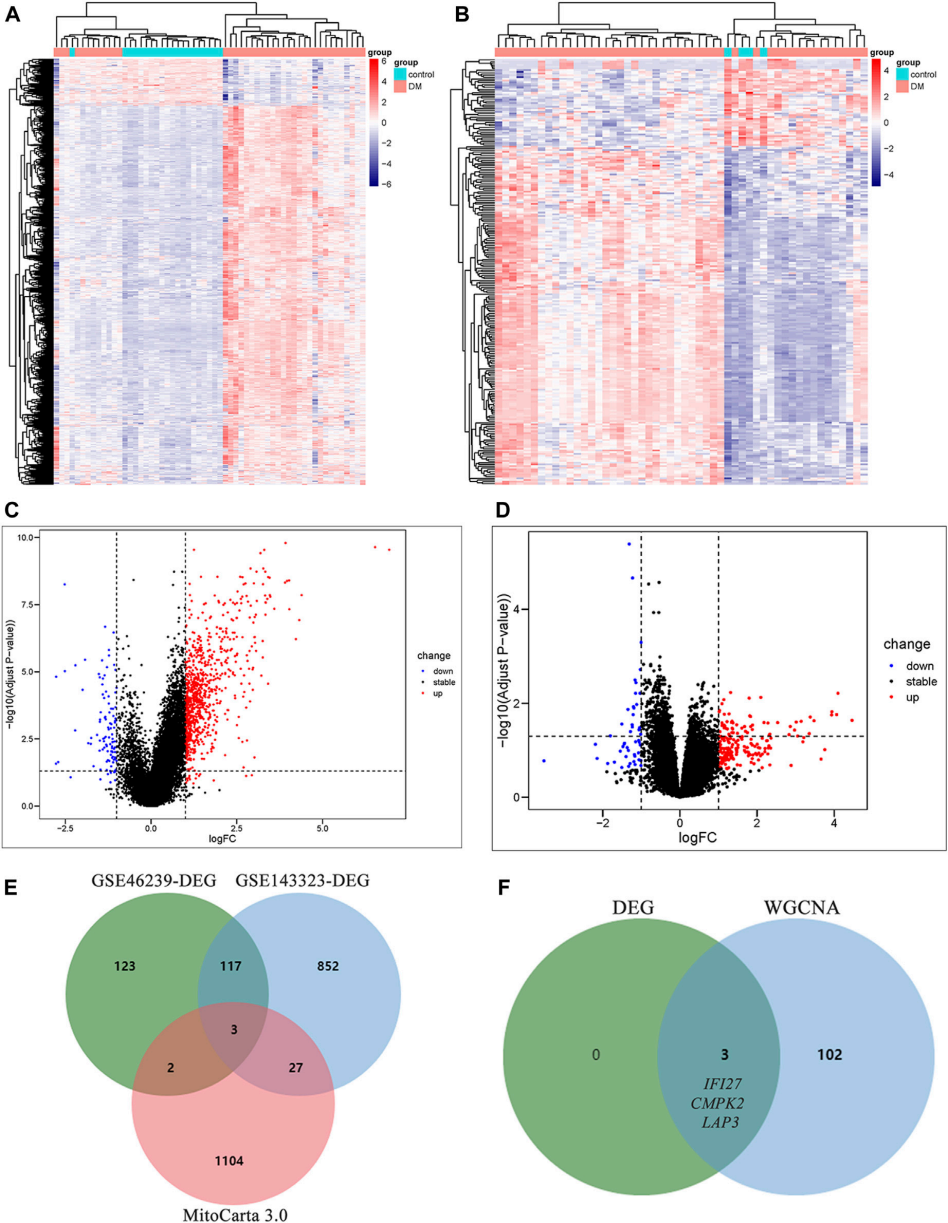
Identification of DEGs in DM and screening of mito-DEGs in muscle and skin tissues from the dataset GSE143323 and GSE46239 via DEG analysis. **(A)** Heatmap of DEGs in GSE143323 dataset (muscle). **(B)** Heatmap of DEGs in GSE46239 dataset (skin). **(C)** Volcano plot of DEGs in GSE143323 dataset (muscle). **(D)** Volcano plot of DEGs in GSE46239 dataset (skin). **(E)** Venn diagram showing the intersections of genes among muscle DEGs, skin DEGs and MRGs. **(F)** Venn diagram of intersection genes from WGCNA and DEG analysis. DM, dermatomyositis, MRG, mitochondrial-related gene; DEGs, differentially expressed genes.

Differential gene expression analysis was also conducted on GSE143323 and GSE46239 datasets. In the GSE143323 dataset, a total of 999 DEGs were identified, 899 of which were upregulated and 110 of which were downregulated (Figures 4A, C). Similarly, in the GSE46239 dataset, 245 DEGs were identified, with 195 genes upregulated and 50 genes downregulated (Figures 4B, D). The DEGs from both datasets were visualized with heatmaps and Volcano plots, providing a comprehensive overview of their expression patterns (Figures 4A–D). Using a Venn diagram, the DEGs in GSE143323 and GSE46239 were then intersected with 1,136 MRGs and yielded three common mito-DEGs (Figure 4E). Furthermore, enrichment analysis demonstrated significant enrichment of pathways associated with “nucleotide metabolism,” “amino acid metabolism,” and “glutathione metabolism,” which aligns with the findings of the WGCNA analysis (Figures 3C, D).

### 3.3 DM-mito hub genes screening and validation in DM

**FIGURE 5:**
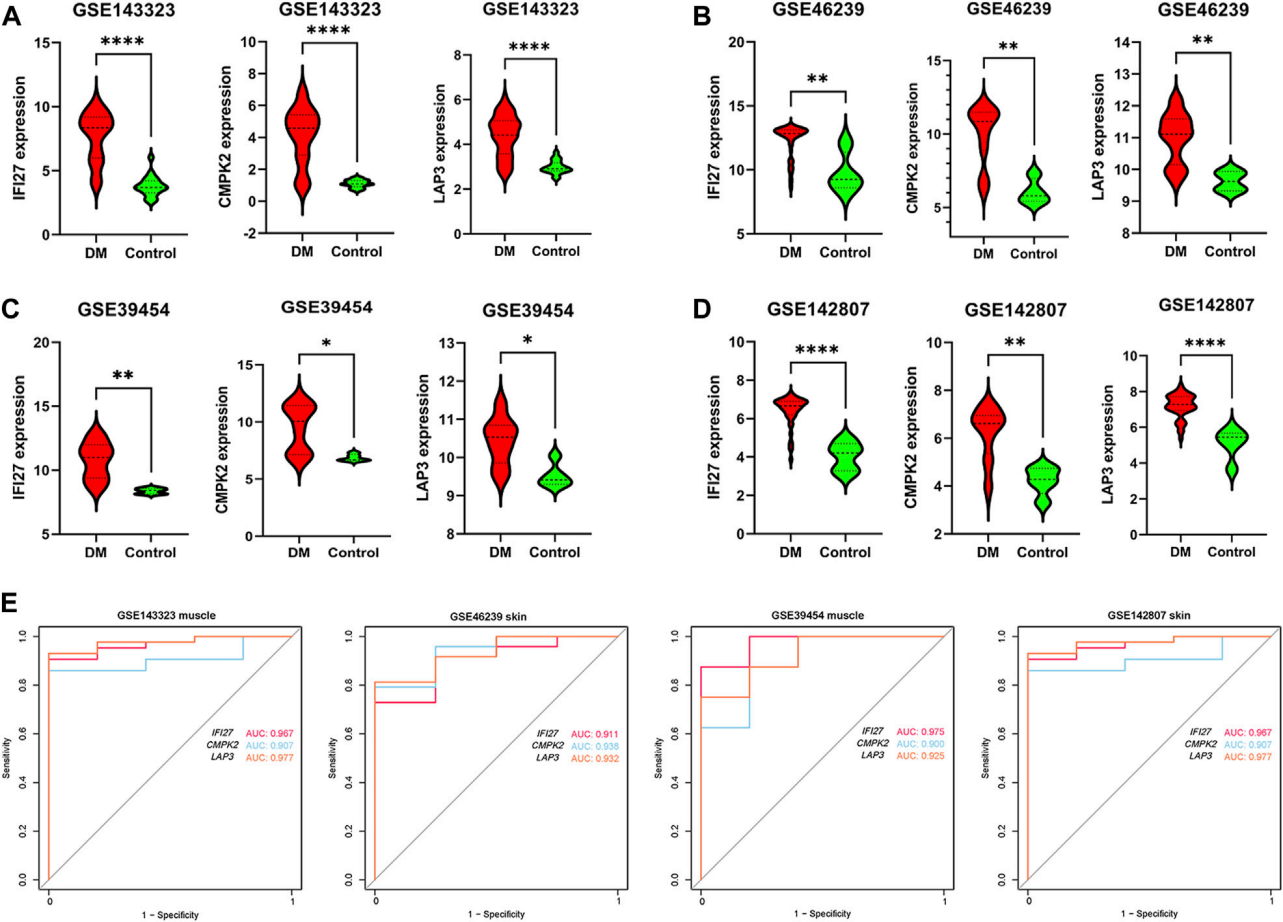
Verification of expression pattern and diagnostic capacity. **(A)** Comparison of *IFI27*, *CMPK2* and *LAP3* expression levels between DM (*n* = 39) and healthy controls (*n* = 20) in dataset GSE143323 (muscle). **(B)** Comparison of *IFI27*, *CMPK2*, and *LAP3* expression levels between DM (*n* = 48) and healthy controls (*n* = 4) in dataset GSE46239 (skin). **(C)** Comparison of *IFI27*, *CMPK2* and *LAP3* expression levels between DM (*n* = 8) and healthy controls (*n* = 5) in dataset GSE39454 (muscle). **(D)** Comparison of *IFI27*, *CMPK2* and *LAP3* expression levels in DM (*n* = 43) and healthy controls (*n* = 5) in dataset GSE142807 (skin). **(E)** The diagnostic value of *IFI27*, *CMPK2* and *LAP3* in the four datasets. *p < 0.05; **p < 0.01; ****p < 0.0001.

**FIGURE 6:**
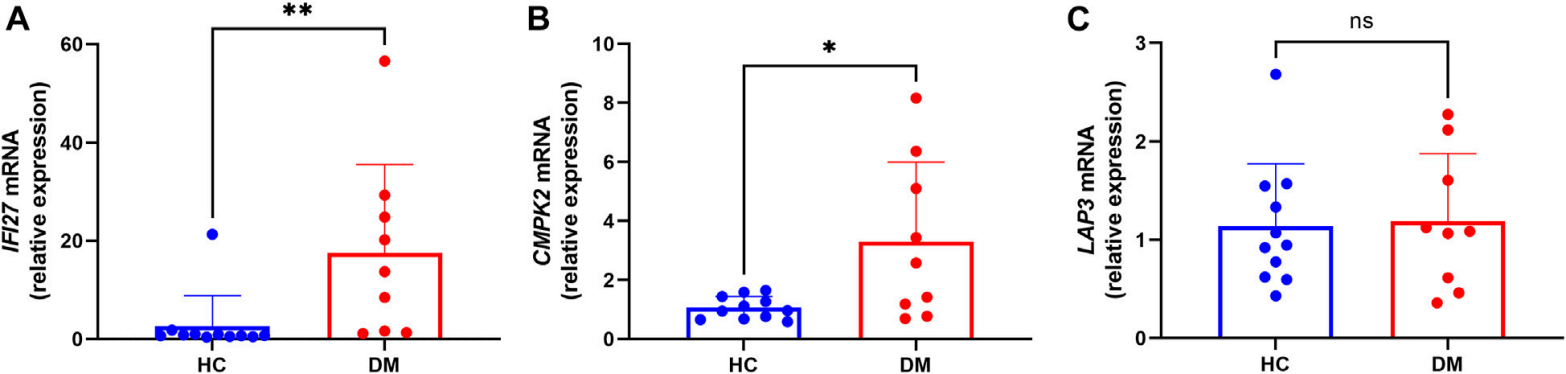
Transcription levels of the *IFI27* **(A)**, *CMPK2* **(B)** and *LAP3* mRNA **(C)** (DM group n = 9; HC group n = 11).

**FIGURE 7:**
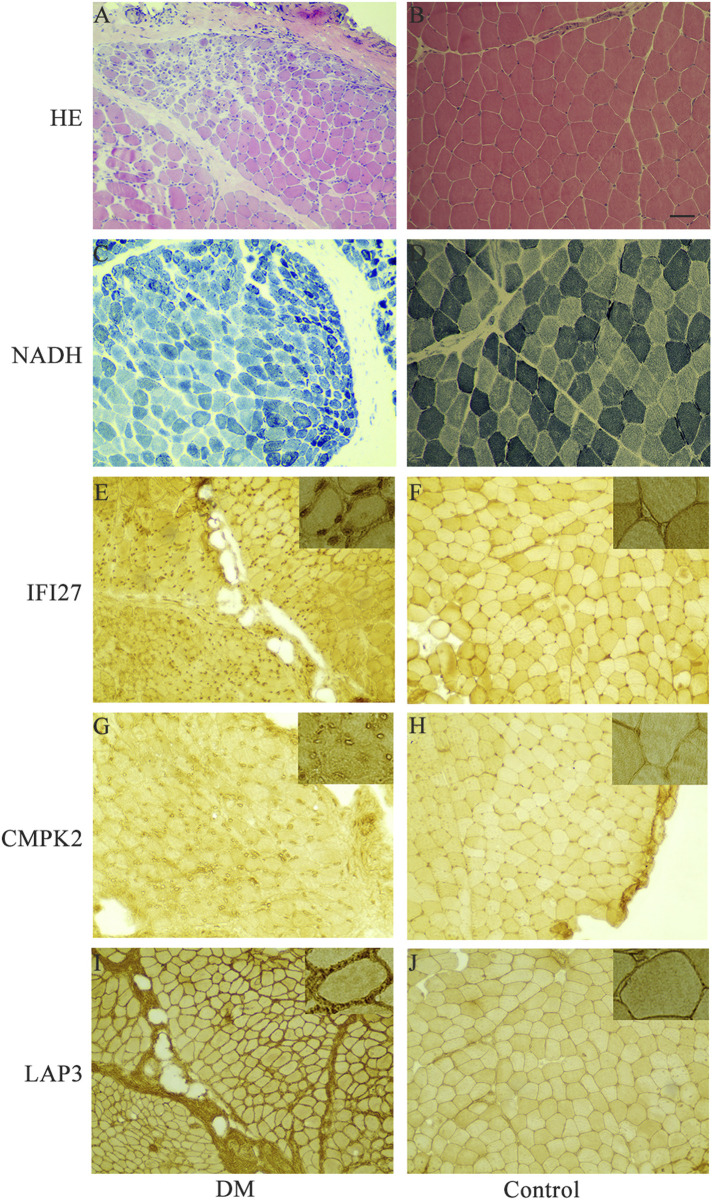
Representative histochemical and immunohistochemical staining in muscle tissues of DM patients and control group. **(A, B)** Muscle sections from DM and controls were subjected to Haematoxylin and Eosin (H&E) staining. **(C, D)** NADH tetrazolium reductase (NADH-TR) staining was performed on DM and control muscle sections. **(E, F)** Immunohistochemistry using anti-human IFI27 antibody on DM and control samples. **(G, H)** Immunohistochemistry using anti-human CMPK2 antibody on DM and control samples. **(I, J)** Immunohistochemistry using anti-human LAP3 antibody on DM and control samples. Insets in the upper right corner represent a 400x magnification. Scale bars = 100 μm. DM, dermatomyositis.

In order to identify mitochondrial-related hub genes in DM, we performed an intersection analysis between mito-MGs obtained from WGCNA analysis and the mito-DEGs from the DEG sets. As a result, three DM-mito hub genes, namely, *IFI27*, *CMPK2*, and *LAP3*, were identified (Figure 4F). Two independent datasets, GSE39454 and GSE142807, were used to validate the expression levels of these hub genes. Remarkably, in both muscle and skin tissues, all hub genes were significantly upregulated compared to the healthy control group (Figures 5A–D). Additionally, the diagnostic accuracy of three hub genes was also assessed across two muscle datasets and two skin datasets. Encouragingly, all datasets exhibited area under the curve (AUC) values >0.9, demonstrating their strong potential as diagnostic markers for DM (Figure 5E). However, only relative molecular expression of *CMPK2* (*Z* = −2.089, *p* = 0.038) and *IFI27* (*Z* = −3.077, *p* = 0.001) was found to be significantly upregulated after peripheral blood validation, *LAP3* was not differentially expressed between DM and control group (*Z* = −0.266, *p* = 0.824) (Figures 6A–C). Furthermore, histological staining was performed on muscle tissues from both DM patients and the control group. As depicted in Figures 7A–D, pronounced perifascicular atrophy was observed in DM patients, accompanied by abnormal NADH-TR staining within atrophic muscle fibers, indicative of mitochondrial dysfunction in the muscle tissues of DM patients. Moreover, immunohistochemical staining revealed a substantial upregulation of IFI27, CMPK2, and LAP3 proteins in the muscle tissues of DM patients (Figures 7E–J). Notably, the expressions of IFI27 and CMPK2 exhibited marked elevation within the nuclei of perifascicular muscle fibers (Figures 7E–H), while LAP3 demonstrated predominant overexpression in the cell membrane and cytoplasm in DM patients (Figures 7I, J).

### 3.4 Immune infiltration in DM and its correlation with DM-mito hub genes

**FIGURE 8:**
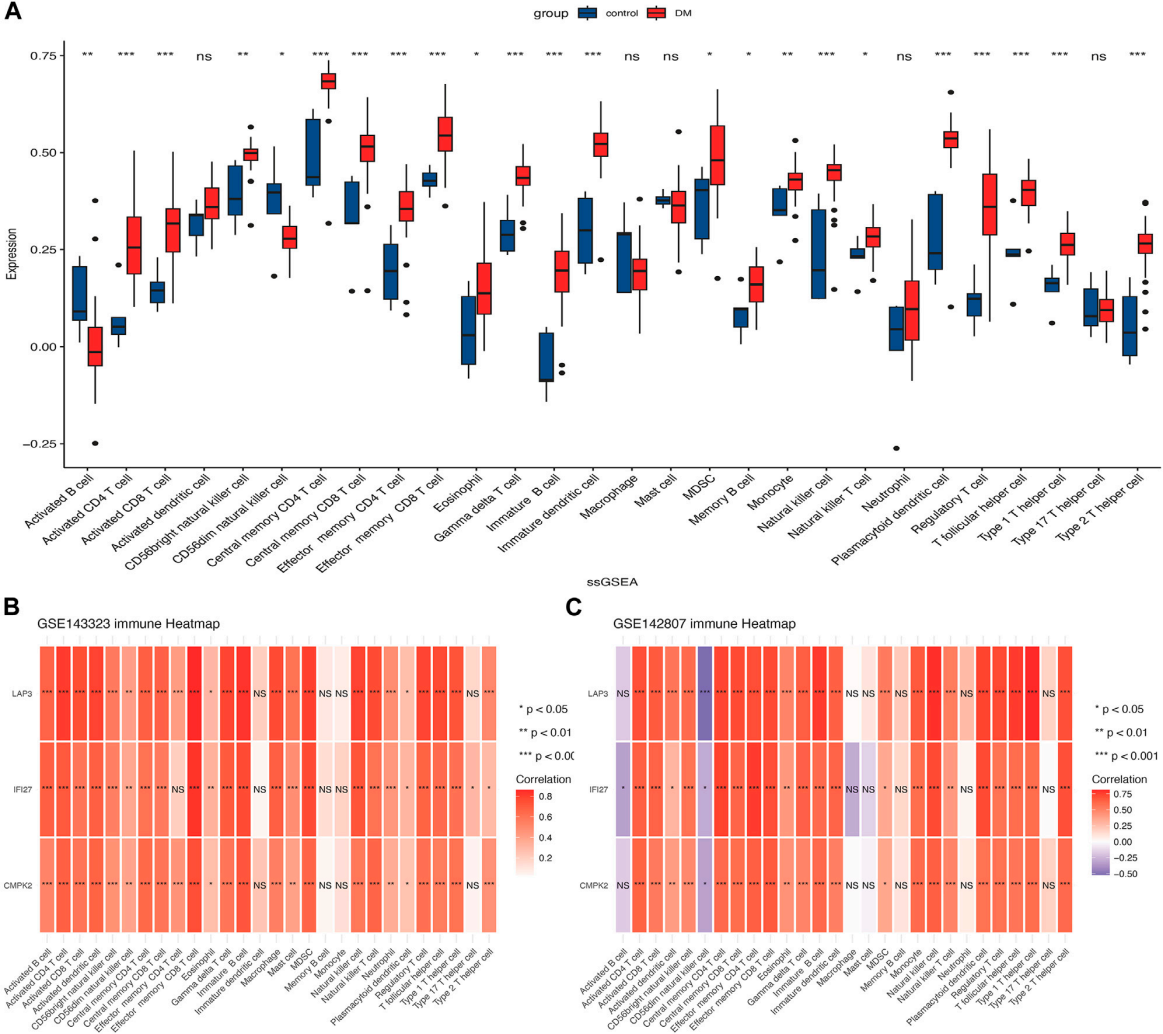
Analysis of immune cell infiltration in muscle and skin tissues of dermatomyositis (DM). **(A)** The distribution of 28 types of immune cells in the GSE142807 dataset using the ssGSEA algorithm. **(B)** The association heatmap of hub genes and immune landscape in the GSE143323 dataset. **(C)** The association heatmap of hub genes and immune landscape in the GSE142807 dataset. *p < 0.05; **p < 0.01; ***p < 0.001; ns = non-significance. DM, dermatomyositis.

The ssGSEA algorithm was employed to evaluate the degree of 28 immune cell infiltration in both muscle and skin tissues of DM. As depicted in Figure 8A, and our previous published findings (reference: The molecular mechanism underlying dermatomyositis-related interstitial lung disease: evidence from bioinformatic analysis and in vivo validation) it revealed analogous immune infiltration patterns between the two tissues, characterized by the presence of activated CD4 T cells, CD8 T cells, Gamma delta T cells, regulatory T cells, T follicular helper cells, and dendritic cells (DC). Notably, there was a positive correlation between hub genes and immune cell abundance (Figures 8B, C).

### 3.5 Prediction of TF-hub gene regulatory network

**FIGURE 9:**
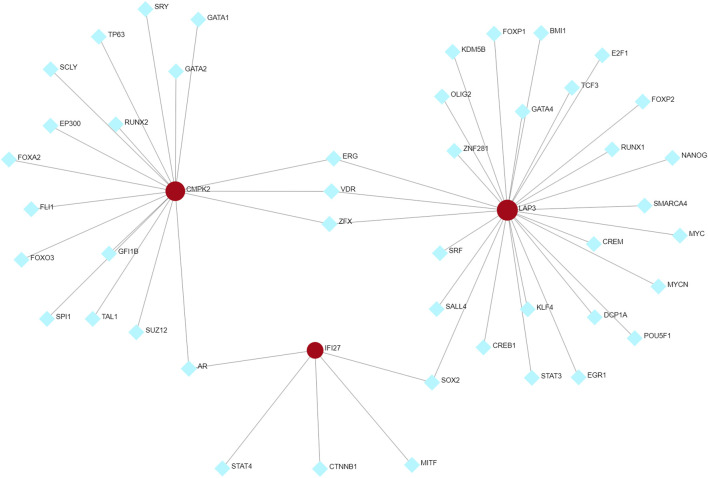
TFs-hub genes regulatory network. Hub genes are indicated by the red circle, while TFs are showed by the blue diamond. TFs, transcription factors.

We used Networkanalyst to predict possible interactions between the three hub genes and TFs. The resulting TFs-genes regulatory network was visualized using Cytoscape. It revealed significant interactions involving ERG, VDR, and ZFX with *CMPK2* and *LAP3*, as well as interactions involving SOX2 with *LAP3* and *IFI27*, and AR with *IFI27* and *CMPK2* (Figure 9). These findings suggest their potential regulatory influence of these TFs on the expression level of the hub genes. However, further research is necessary to validate these observations.

## 4 Discussion

DM is an inflammatory myopathy that affects various organs, including the muscles and skin (Lundberg et al., 2021). Extensive research has been conducted to understand the underlying mechanisms of DM, revealing the significance of mitochondrial dysfunction in its pathogenesis (Alhatou et al., 2004; Varadhachary et al., 2010; Gonzalez-Chapa et al., 2023). However, the specific pathogenic mechanism of mitochondrial dysfunction in DM remains unelucidated. In our study, we integrated GEO database and used bioinformatics and experimental methods to investigate the mechanisms of mitochondrial dysfunction in DM pathogenesis.

Genes within MEturquoise and MEmagenta were intersected with a set of MRGs, resulting in 105 mito-MGs. Potential functional mining of these genes using GO and KEGG analysis suggests that they are closely linked to OXPHOS and energy metabolism. Since mitochondria are the primary sites of bioenergy metabolism, their abnormal pathological changes have been detected in muscle fibers of DM patients (Varadhachary et al., 2010). Furthermore, studies have also reported abnormal expression of MRGs in DM patients (Lax et al., 2011; Meyer et al., 2017). Meyer et al. (2017) found downregulation of genes involved in encoding electron transport chain complex proteins (e.g., NDFAB11, COX8a, and ATP5D) and proteins associated with mitochondrial biogenesis (TFAM and PPARGC1A) in muscle biopsies from DM patients compared to those from healthy individuals, while genes promoting transcript levels of type I IFNs were upregulated (Lax et al., 2011; Bolko et al., 2021; Lepelley et al., 2021). Disruption in mitochondrial integrity or compartmentalization can cause mitochondrial nucleic acid to leak into the cytosol, inducing the expression of IFN-related gene (Lepelley et al., 2021). Furthermore, elevated levels of IFN-β expression in the bloodstream can induce muscle mitochondrial dysfunction through increased production of reactive oxygen species (ROS), creating a feedback loop for disease progression and sustaining the disease (Meyer et al., 2017; Duvvuri et al., 2023). In addition, DM patients exhibit significantly higher levels of H_2_O_2_ production compared to normal controls, indicating increased ROS levels due to mitochondrial dysfunction (Meyer et al., 2017). Respiratory chain dysfunction and reduced OXPHOS are observed in DM, particularly in perifascicular regions (Alhatou et al., 2004; Hedberg-Oldfors et al., 2022), and Carola et al. found a strong association between depletion of mtDNA and respiratory chain dysfunction in DM muscle (Hedberg-Oldfors et al., 2022). Therefore, the deterioration of muscle tissue in patients with DM may be closely associated with mitochondrial dysfunction.

Next, we performed an intersection analysis of mito-MGs and mito-DEGs and identified three DM-mito-hub genes (*IFI27*, *CMPK2*, and *LAP3*). Using RT-qPCR, the hub genes *IFI27* and *CMPK2* were confirmed to have consistent expression patterns with the datasets, while *LAP3* did not show significant differences. Furthermore, immunohistochemical staining revealed a significant upregulation of IFI27, CMPK2, and LAP3 proteins in the muscle tissues of DM patients, especially prominent in the perifascicular region. The lack of significant difference in qPCR results of *LAP3* could be due to several factors. First, the samples for bioinformatic analysis were derived from muscle and skin tissues, while peripheral blood samples were used for the qPCR validation. Gene expression levels in blood samples do not always directly correlate with protein levels in different tissues. The gene expression analysis using qPCR in blood samples may capture the transcriptional changes occurring at a systemic level, reflecting the overall gene activity related to DM. On the other hand, the elevated protein levels of all three genes in muscle tissues may signify localized effects specific to the affected tissue in the context of DM pathogenesis. Second, it is important to consider that the small sample size used for validation may be influenced by individual specificity.

*IFI27*, a type I IFN-regulated genes, exerts upstream or internal negative regulation on RNA polymerase II transcription and nucleoprotein export (Swadling et al., 2022). It also plays a significant role in mitochondrial metabolism. Additionally, IFI27 physically links the succinate dehydrogenase complex iron sulfur subunit B (SDHB) to the chaperone TNF receptor associated protein 1 (TRAP1), protecting SDHB from oxidative damage-triggered degradation. Furthermore, IFI27 enhances the catalytic activity of hydroxyacyl-CoA dehydrogenase trifunctional multienzyme complex subunit alpha (HADHA) in the β-oxidation pathway (Cui et al., 2023). Elevated expression of IFI27 has been linked to immune responses and mitochondrial dysfunction (Hsiao et al., 2013). Support these findings, increased transcript levels of *IFI27* were observed in deltoid tissue of DM patients (Meyer et al., 2017). The *CMPK2* gene, located in the nuclear genome, encodes a mitochondrial kinase involved in the salvage pathway of deoxyribonucleotide synthesis into organelles. Its deficiency can lead to mitochondrial DNA depletion syndrome (Arumugam et al., 2021), leading to diminished ATP production, and disturbances in mitochondrial cristae architecture (Zhao M. et al., 2022). Studies have shown that *CMPK2,* enriched in mitochondria, increases IFN-α-induced ROS production and inflammasome activation (Lai et al., 2021). Silencing of CMPK2 in macrophages results in augmented ROS and perturbation of mitochondrial structure, leading to the upregulation of pro-inflammatory genes IL1β, TNFα, and IL8 (Arumugam et al., 2022). Studies have reported a significantly increase of *CMPK2* expression in active SLE compared to healthy individuals and inactive SLE patients, making it a promising biomarker for diagnosis and evaluation of SLE activity (Xu et al., 2008; Li et al., 2022). Leucine aminopeptidase 3 (LAP3), encoded by the *LAP3* gene, exhibits dual subcellular localization in both the cytosol and mitochondria (Sickmann et al., 2003). LAP3 plays a key role in cleaving amino acids from the N-terminus of peptides and proteins, essential for regulating protein degradation and peptide metabolism (Li et al., 2022). Notably, LAP3 has been found to influence muscle development, with its silencing significantly impeding muscle fiber formation and heightened LAP3 expression markedly promoting muscle fiber formation in a sheep model (Ge et al., 2022). Furthermore, LAP3 contributes to the depletion of arginine induced by IFN-γ in a bovine mammary epithelial cell model (Li et al., 2022). While only one study has identified an upregulation in LAP3 expression within DM (Chen et al., 2022), the exact underlying mechanism remains to be elucidated. In DM, a disease with significant mitochondrial dysfunction, patients exhibit a notable reduction in aerobic capacity with histopathological and biochemical evidence supporting the presence of OXPHOS dysfunction, particularly in the perifascicular regions (Woo et al., 1988a; Cea et al., 2002; Alhatou et al., 2004; Varadhachary et al., 2010; Meyer et al., 2017). It is speculated that the widely acknowledged crucial pathway in DM triggers the increase in *IFI27*, *CMPK* and *LAP3* expression, potentially exacerbating mitochondrial dysfunction and augmenting the production of ROS and inflammation in DM.

Patients with DM are in greater risk of developing cancer than in the general population, with cancer incidence peaking 1 year after disease diagnosis (Yang et al., 2015). Ovarian, lung, and breast cancers as well as non-Hodgkin lymphoma, are common cancers in DM, followed by cancers of the digestive and urinary systems (Hill et al., 2001; Jakubaszek et al., 2015; McGrath et al., 2018). We predicted TFs that might interact with three hub genes using TF hub gene regulatory networks. Among them, ERG, ZFX and SOX2 have been reported to be highly associated with tumor development (Baldus et al., 2006; Jahanimoghadam et al., 2022; Schmoellerl et al., 2023; Wang et al., 2023). The development of tumors in DM patients may be associated with these TFs, however, no relevant reports are available at present, and further investigation is required to confirm and demonstrate this association in the future.

We utilized ssGSEA to investigate immune landscape in DM, encompassing 28 immune cells within muscle and skin tissues. Our analysis identified activated CD4 T cells, CD8 T cells, γδ T cells, regulatory T cells, T follicular helper cells, and dendritic cells in both DM-affected tissues. Previous research has firmly established that the predominant muscular inflammatory infiltrate in DM comprises CD4 T cells, often accompanied by plasmacytoid dendritic cells and B cells (Arahata and Engel, 1984). Helper T cells, a subset of CD4 T cells, played a crucial role in coordinating B cells for antibody regulation (Gensous et al., 2018). In both muscle and peripheral blood of DM patients, T follicular helper (Tfh) cells have been shown to exhibit associations with disease activity, particularly concerning cutaneous manifestations and muscular weakness, and hormone therapy notably reduced peripheral blood helper T cells (Hou et al., 2021). In addition, another study has identified a significant upregulation of IL21, a prominent cytokine primarily synthesized by Tfh cells, within the context of dermatomyositis, thereby further implicating their pivotal role in the pathogenesis of DM (Liu et al., 2017). These findings collectively emphasize T cell immunity dysregulation as a central factor in dermatomyositis pathogenesis, offering potential therapeutic insights. We also observed that three mito-hub genes positively correlated with increased immune cell infiltration in DM. We propose that inflammatory cell infiltration leads to muscle fiber degeneration, necrosis, and mitochondrial dysfunction. Abnormal mitochondria may elevate reactive oxygen species, exacerbating immune activation and perpetuating the disease cycle.

Nevertheless, the study still has a few limitations. The datasets employed for analysis were derived from varying sources and can introduce inherent variability potentially influencing the robustness and generalizability of the findings. The sample size used for validation is small, which might influence the statistical power of our results. Although we used RT-qPCR and immunohistochemistry for validation, functional validation experiments are still needed to further elucidate the precise molecular mechanisms underlying the observed correlations.

## 5 Conclusion

In summary, based on a comprehensive gene expression meta-analysis, we have identified three mito-hub genes (*IFI27*, *CMPK2*, and *LAP3*) that are closely associated with DM pathogenesis, opening new perspectives about the pivotal role of mitochondria in the disease.

## Data Availability

The datasets presented in this study can be found in online repositories. The names of the repository/repositories and accession number(s) can be found in the article.
