# Supplementary material for: Mitochondrial-related hub genes in dermatomyositis: muscle and skin datasets-based identification and in vivo validation
Source: Front Genet. 2024 Feb 8;15:1325035. doi: 10.3389/fgene.2024.1325035 (PMC10882082; doi:10.3389/fgene.2024.1325035)
Supplement: Supplementary file 2 [file Table1.DOCX]

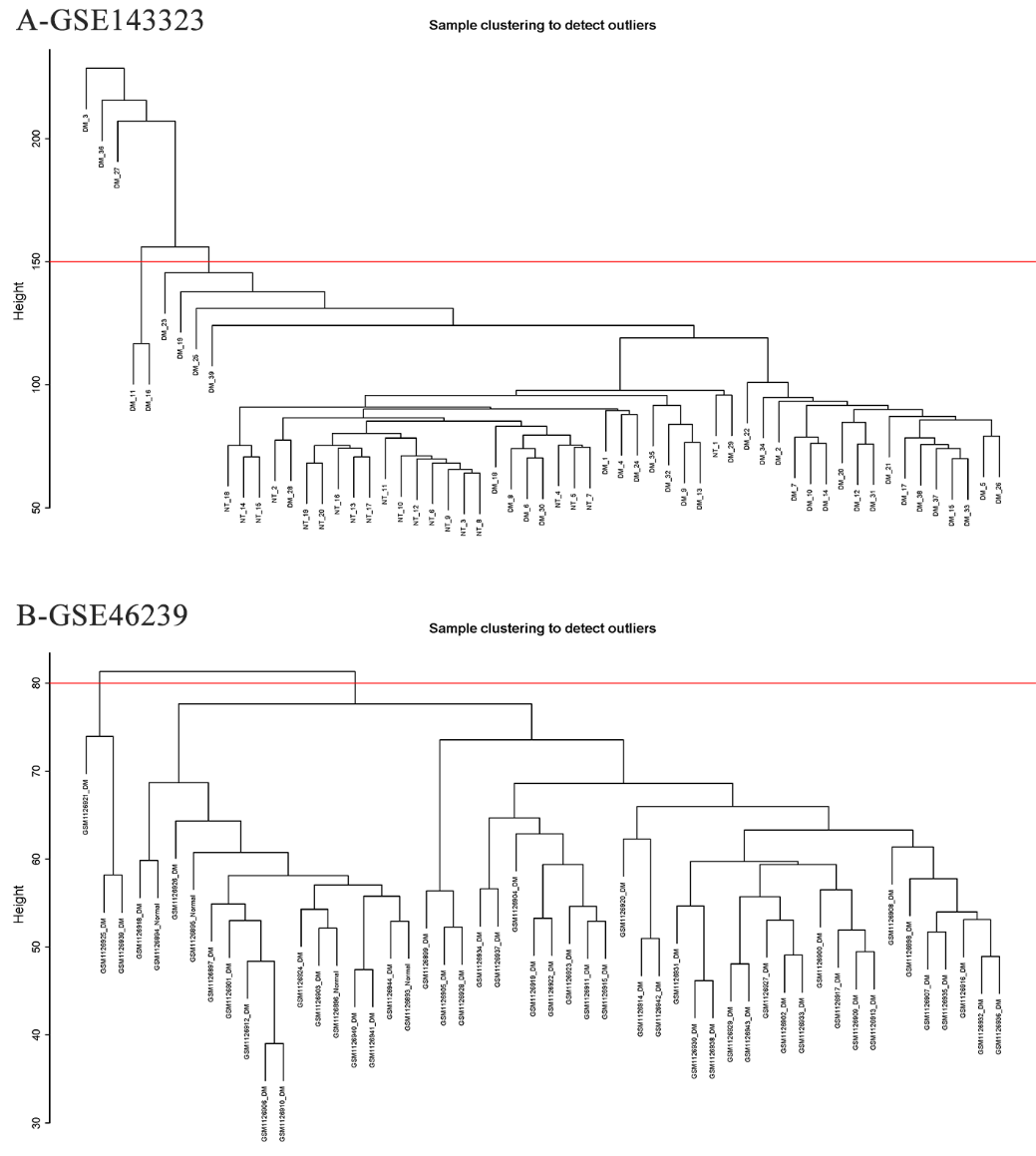


**Supplementary File 1.** Clustering dendrogram of muscle samples (A) and skin tissues (B). The ordinate shows the clustering height while the abscissa displays the name of the samples.
