# Supplementary material for: Mitochondrial-related hub genes in dermatomyositis: muscle and skin datasets-based identification and in vivo validation
Source: Front Genet. 2024 Feb 8;15:1325035. doi: 10.3389/fgene.2024.1325035 (PMC10882082; doi:10.3389/fgene.2024.1325035)
Supplement: Supplementary file 3 [file Table3.docx]

**
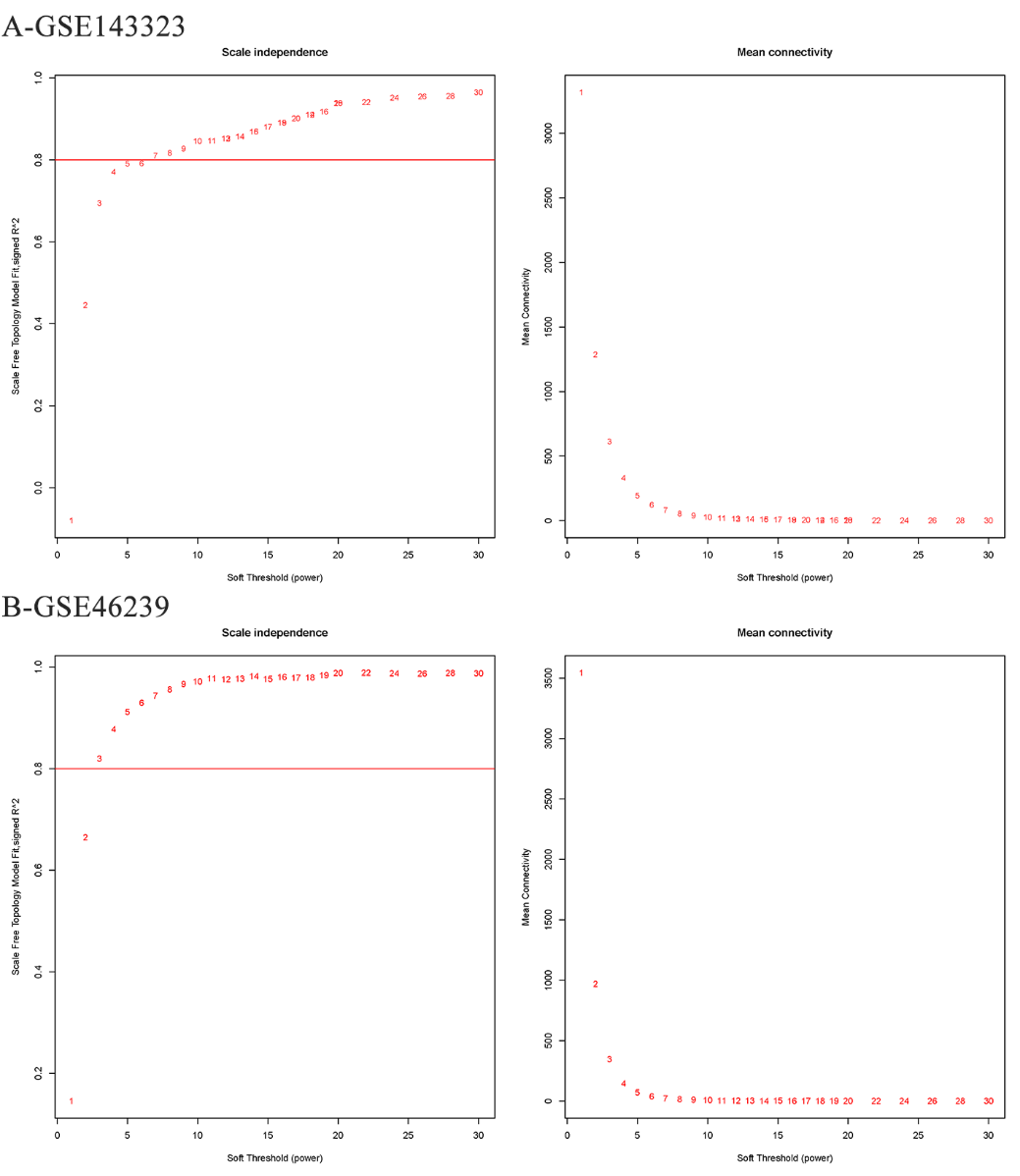
**
**Supplementary File 3.** Determination of soft thresholding power in WGCNA analysis. The red line represents the threshold where the square of the correlation coefficient equals 0.8. In the left panel, the scale-free fit index (scale independence, y-axis) is plotted against the soft threshold power (x-axis). In the right panel, the mean connectivity (degree, y-axis) is plotted against the soft threshold power (x-axis). (A) In the GSE143323 dataset, the soft thresholding power of 7 achieves an approximate scale-free fit index. (B) In the GSE46239 dataset, the soft thresholding power of 3 achieves an approximate scale-free fit index.
